# eyeNotate: Interactive Annotation of Mobile Eye Tracking Data Based on Few-Shot Image Classification

**DOI:** 10.3390/jemr18040027

**Published:** 2025-07-07

**Authors:** Michael Barz, Omair Shahzad Bhatti, Hasan Md Tusfiqur Alam, Duy Minh Ho Nguyen, Kristin Altmeyer, Sarah Malone, Daniel Sonntag

**Affiliations:** 1 Interactive Machine Learning, German Research Center for Artificial Intelligence (DFKI), 66123 Saarbrücken, Germany; omair_shahzad.bhatti@dfki.de (O.S.B.); hasan_md_tusfiqur.alam@dfki.de (H.M.T.A.); ho_minh_duy.nguyen@dfki.de (D.M.H.N.); daniel.sonntag@dfki.de (D.S.); 2 Applied Artificial Intelligence, University of Oldenburg, 26129 Oldenburg, Germany; 3 Machine Learning and Simulation Science Department, University of Stuttgart, 70569 Stuttgart, Germany; 4 Max Planck Research School for Intelligent Systems (IMPRS-IS), 70569 Stuttgart, Germany; 5 Department of Education, Saarland University, 66123 Saarbrücken, Germany; kristin.altmeyer@uni-saarland.de (K.A.); s.malone@mx.uni-saarland.de (S.M.)

**Keywords:** eye tracking, interactive machine learning, area of interest (AOI), mobile eye tracking, visual attention, eye tracking data analysis, fixation-to-AOI mapping

## Abstract

Mobile eye tracking is an important tool in psychology and human-centered interaction design for understanding how people process visual scenes and user interfaces. However, analyzing recordings from head-mounted eye trackers, which typically include an egocentric video of the scene and a gaze signal, is a time-consuming and largely manual process. To address this challenge, we develop eyeNotate, a web-based annotation tool that enables semi-automatic data annotation and learns to improve from corrective user feedback. Users can manually map fixation events to areas of interest (AOIs) in a video-editing-style interface (baseline version). Further, our tool can generate fixation-to-AOI mapping suggestions based on a few-shot image classification model (IML-support version). We conduct an expert study with trained annotators (n = 3) to compare the baseline and IML-support versions. We measure the perceived usability, annotations’ validity and reliability, and efficiency during a data annotation task. We asked our participants to re-annotate data from a single individual using an existing dataset (n = 48). Further, we conducted a semi-structured interview to understand how participants used the provided IML features and assessed our design decisions. In a post hoc experiment, we investigate the performance of three image classification models in annotating data of the remaining 47 individuals.

## 1. Introduction

Eye tracking studies often consider visual attention to specific areas of interest (AOIs) to analyze and understand how people process visual information. AOIs are specific regions in a scene or interface that are defined by researchers [1]. Visual attention refers to the time a person pays attention to these regions. By measuring visual attention to and transitions between AOIs during a study, researchers can gain insights into which elements of a scene are relevant to an activity and how interventions of an experiment influence the participant’s eye movement behavior. This is usually performed based on fixation events as they are assumed to approximate a person’s allocation of cognitive resources through the time they spend processing a visual scene [2]. Further, advances in modern head-worn eye tracking technology [3] can enable attention-aware mobile human–computer interfaces. In remote eye tracking with static stimuli such as images, an AOI can be defined once and reused for every participant. Dynamic AOIs in video-based stimuli can be annotated using keyframe-based annotation techniques; i.e., AOIs are marked via bounding boxes for keyframes, and interpolation is used to annotate intermediate frames [4]. However, these efficient fixation-to-AOI mapping techniques from remote eye tracking do not scale for mobile eye tracking applications. Accurately annotating mobile eye tracking data remains a challenging and time-consuming task because scene videos taken with a head-mounted eye tracking device are unique for every participant. In mobile eye tracking practice, one or more annotators decide per fixation whether an AOI was hit or not [5,6]. This fixation-wise annotation approach reduces the annotation effort compared to a video frame-based annotation because fixations last around 200–400 ms [1], which corresponds to 2–2.5 events per second. Videos are typically recorded with a sampling rate of at least 30 Hz. Still, it does not remedy the need to annotate AOIs in every single recording and hinders the development of attention-aware mobile interfaces.

Attaching fiducial markers to target stimuli was proposed as a solution in research [7,8,9] and was adopted in modern commercial software solutions like Pupil Cloud (https://pupil-labs.com/blog/pupil-cloud-projects-enrichments/; accessed on 2 February 2024). However, markers are obtrusive and may impact visual scanning behavior. Therefore, the present research aims at a solution for non-instrumented environments. Existing approaches for automatic or semi-automatic analysis of head-mounted eye tracking data use computer vision models to map fixations to AOIs. Most of these approaches rely on pre-trained computer vision models that do not allow for adapting the underlying model to a certain target domain [5,10,11,12,13]. These can be applied in very constrained settings only, i.e., if the dataset used for training the machine learning model matches the target domain. Some approaches support a single, a priori model training or fine-tuning step for adaptation to a target domain [14,15,16]. These approaches offer no possibility of adapting the model during the annotation process and, hence, suffer from a lack of flexibility. Further, not all methods are evaluated quantitatively [17,18,19] or evaluation metrics are not properly described [11,20] or inadequate, e.g., ignoring temporal aspects [16]. Some commercial tools offer automatic mapping of the gaze signal in world video coordinates to a reference frame that defines AOIs, such as the assisted mapping function of Tobii Pro (https://connect.tobii.com/s/article/how-to-perform-manual-and-assisted-mapping; accessed on 12 December 2024). However, this is only possible for a limited number of reference frames.

We aim to develop a method for semi-automatic mapping of fixations to AOIs, which enables efficient analysis and interpretation of humans’ complex interaction behavior. This bears the potential to boost the efficiency in research based on eye tracking by automating the time-consuming and expensive data annotation process [16] and to facilitate novel real-time adaptive human–computer interaction [21,22]. Further, we aim to break the limitations of using pre-trained models, i.e., the issue of lacking flexibility and quality assurance through humans-in-the-loop. In this work, we implement and evaluate eyeNotate, a user interface that enables semi-automatic annotation of mobile eye tracking data. Our tool allows mobile eye tracking practitioners to manually annotate their recordings fixation-wise, reflecting the current state of the art and representing our baseline approach. Further, we implement an extension offering fixation-to-AOI mapping suggestions using a few-shot image classification model, which was shown to be successful in another use case [23]. This model can learn from user feedback, i.e., when users accept or reject/correct suggestions, following the interactive machine learning (IML) paradigm. IML combines frequent human input and feedback with machine learning technologies without requiring background knowledge in machine learning [24,25]. Domain knowledge from end-users, like eye tracking practitioners, can be integrated more effectively into complex applications. However, it is important to thoroughly design such systems to achieve better user experiences and more effective learning systems [26]. We conduct a case study with n = 3 trained annotators to compare the baseline version and the IML-supported approach. We measure the perceived usability, annotation validity and reliability, and efficiency during a data annotation task using an existing mobile eye tracking dataset with ground-truth annotations (n = 48). We ask participants to re-annotate data for one individual in this dataset. After task completion, we conducted a semi-structured interview (SSI) to understand how participants used the provided IML features. In addition, we investigate the performance in automatically annotating the remainder of the dataset using our resulting machine learning models.

To address the challenges in annotating data from head-mounted eye trackings, we implement eyeNotate, a user interface that enables semi-automatic annotation. Our tool allows mobile eye tracking practitioners to manually annotate their recordings fixation-wise (baseline) and semi-automatically using fixation-to-AOI mapping suggestions based on a few-shot image classification model (IML-support). We contribute by (i) implementing the eyeNotate tool for semi-automatic annotation of head-mounted eye tracking data based on few-shot image classification, (ii) evaluating our eyeNotate in a case study with n = 3 trained annotators to compare the baseline version and the IML-supported approach, measuring the perceived usability, annotation validity and reliability, and efficiency during a data annotation task, and (iii) conducting a post hoc machine learning experiment to assess the performance of the considered models in automatically annotating data from head-mounted eye trackers.

## 2. Related Work

We aim to improve the annotation process for mobile eye tracking data from diagnostic user studies, i.e., assigning each fixation in a set of recordings to an AOI based on the corresponding video frame from the front-facing scene camera and the fixation position. Here, we provide an overview of existing approaches for the annotation of mobile eye tracking data and video annotation in general. Further, we provide a brief overview of methods for real-time interpretation of eye tracking data that can be used to develop wearable attention-aware user interfaces [27]. Using unobtrusive modern eye tracking head-gear (see, e.g., Tonsen et al. [3], Lander et al. [28]) or augmented reality headsets like Microsoft’s HoloLens 2 that come with integrated eye tracking sensors, our system for interactive annotation and model training can enable developers to easily create custom computer vision models for attention-aware mobile interaction.

### 2.1. Annotation of Data from Mobile Eye Trackers

Head-mounted eye trackers allow researchers to investigate human behavior in mobile settings. However, efficient methods for mapping fixations to AOIs from remote eye tracking cannot be used because the video of the front-facing scene camera differs for each participant. Instrumenting the experiment scene with fiducial markers is an option to cope with this issue [7,8]. Software that accompanies modern head-mounted eye trackers typically integrates marker tracking, like the marker-based surface tracking in Pupil Capture [29]. However, the instrumentation of the experiment area comes with certain limitations. Marker tracking might be lost due to low camera quality or due to occlusion through other objects in the scene. In augmented reality (AR) settings, which allow learners to see digital objects embedded in reality by looking through the camera of smartphones or tablets, supposedly unique markers might appear twice, causing ambiguity. Consequently, objects can no longer be distinctly identified by markers. Another disadvantage of marker-based surface tracking is that the numerous markers needed to reliably recognize objects in information-rich learning environments might impair the instructional design by claiming cognitive resources for the marker processing and distracting from learning-relevant visual stimuli. Therefore, this work focuses on an approach to facilitate and support the time-consuming and challenging procedure of mapping human gaze or fixations to objects or AOIs in non-instrumented environments. Commercial tools like Tobii Pro Lab (https://connect.tobii.com/s/article/how-to-perform-manual-and-assisted-mapping (accessed on 12 December 2024)) exist that offer automatic mapping of the gaze signal to AOIs defined in a reference image. However, the assisted mapping function works for static scenes only, is error-prone in cases of fast head movements and distorted image frames, and, hence, requires additional manual effort for correcting wrong assignments or annotating missing samples [15]. Further, the software is very expensive and does not support the annotation of eye tracking data from other devices like Pupil Core head-worn device that we used. Previous research also addressed this problem in the context of data analysis for diagnostic eye tracking studies. However, these approaches come with certain limitations.

Most approaches rely on pre-trained computer vision models that do not support an adaptation of the underlying models to the target domain. Sümer et al. [10] investigated the problem of automatic attention detection in a teaching scenario. They extract image patches for all student faces in the egocentric video feed and cluster them using a ResNet-50 model [30] trained on VGGFace2 data [31]. They assign student IDs to each cluster, allowing them to map the teacher’s gaze to individual students. Chong et al. [32] developed a system for measuring eye contact in adult–child social interactions using mobile eye trackers. Callemein et al. [33] presented a system for detecting when the participant’s gaze focuses on the head or hands of another person without the possibility of differentiating between interlocutors. Machado et al. [11] matched fixations with bounding boxes from an object detection algorithm. They used a sliding-window approach with a MobileNet model [34], pre-trained on ImageNet data [35]. Venuprasad et al. [13] used unsupervised clustering with gaze and object locations to detect visual attention to an object or a face. They used a Faster-RCNN model [36], pre-trained using the MS COCO dataset [37]. Barz and Sonntag [38] compared two approaches for automatic fixation-to-AOI mapping using pre-trained deep learning models: two ResNet models pre-trained with ImageNet data and a Mask R-CNN model pre-trained using MS COCO data. In an evaluation based on the VISUS dataset [6], they found that pre-trained models have severe drawbacks in realistic scenarios like AOIs not being represented by the training data. Deane et al. [12] also presented an annotation system based on a pre-trained Mask R-CNN model [39]. They found high agreements between manual and automatic annotations for AOIs that match the MS COCO classes. These can be applied in very constrained settings only, i.e., if the dataset used for training the machine learning model matches the target domain.

Other approaches suffer from a lack of flexibility. Wolf et al. [14] developed an algorithm that maps fixations to object-based AOIs using the Mask R-CNN object detection model [39]. They conducted a controlled lab study to record data in a healthcare setting with two AOIs: a bottle and five syringes. An evaluation has shown that using 72 training images with 264 annotated object masks, their system can closely approximate the AOI-based metrics compared to manual fixation-wise annotations as a baseline. Batliner et al. [40] presented a similar system for simplifying usability research with mobile eye trackers for medical screen-based devices. Kumari et al. [15] investigate the effectiveness and efficiency of three object detection models for annotating mobile eye tracking data from students participating in STEM lab courses. These methods are based on a single, a priori model training or fine-tuning step with no possibility of adapting the model during the annotation process.

Some approaches include promising interaction concepts but use outdated computer vision methods. Pontillo et al. [20] presented SemantiCode, an interactive tool for post hoc fixation-based annotation of egocentric eye tracking videos. It supports semi-automatic labeling using a distance function over color histograms of manually annotated fixations. Brône et al. [19] proposed to use object recognition with mobile eye tracking to enhance the analysis of customer journeys. In follow-up work, they compared different feature extraction methods [41] and evaluated their approach in a museum setting [42]. Evans et al. [43] reviewed methods for mobile eye tracking in outdoor scenes ranging from pupil detection and calibration to data analysis. They presented an early overview of methods for automating the process of analyzing mobile eye tracking data. Fong et al. [44] presented a semi-automatic data annotation approach. An annotator assigns video frames with a gaze overlay to AOIs, and as the annotation process advances, the system learns to classify AOIs via instance-based learning. Kurzhals et al. [18] used bag-of-SIFT features and color histograms with unsupervised clustering to sort fixation-based image patches by their appearance. They offer an interactive visualization for manual corrections. Panetta et al. [16] presented an annotation method based on bag-of-visual words as features and a support vector classification model (SVC) that is trained a priori. In follow-up work, they present a system that automatically segments objects of interest using two state-of-the-art neural segmentation models [45]. They used pre-trained models to showcase and evaluate new data visualization methods, but they did not assess the performance of their automatic annotation approach.

Recently, Kurzhals et al. [46] described an interactive approach for annotating and interpreting egocentric eye tracking data for activity and behavior analysis. They implement an iterative time sequence search based on eye movements and visual features. They aim to annotate high-level activity events instead of AOI-hit events like we do. In follow-up work, Kurzhals [47] presented an approach for annotating the objects viewed by study participants wearing mobile eye trackers. They propose to crop image patches around each point of gaze, segment the resulting image patches similar to the fixation detection method by Steil et al. [48], and present representative gaze thumbnails to annotators as image clusters in 2D. Annotators interact with this cluster representation to annotate and analyze the mobile eye tracking data. In contrast, our method is based on interactive few-shot image classification. Our system learns to recognize the type of fixated objects or regions based on human feedback during the interaction.

This work aims to accelerate and objectify research on visual attention with mobile eye tracking using technologies from the field of computer vision and interactive machine learning.

### 2.2. Video Annotation in General

The annotation of mobile eye tracking data requires the interpretation of the video feed from the front-facing scene camera. Hence, systems and methods for video annotation are closely related to our approach. An important difference is that general tools for video annotation do not take the gaze signal or fixation events into account. In fact, video annotation based on the definition of bounding boxes around relevant objects, a respective interpolation for intermediate frames, and a mapping of gaze or fixation points to these areas is the state of the art for annotating video stimuli used with remote eye tracking devices [4]. Even though these methods do not scale when it comes to the annotation of mobile eye tracking with individual video feeds for each participant, we briefly review recent approaches and tools for video annotation, as they can provide guidance for the design of similar systems. With LabelMovie, Palotai et al. [49] presented a tool for collaborative video annotation. They proposed machine learning-based quality assurance and automation of the annotation process. In more recent work, the research group presented a method for the semi-automatic annotation of videos for analyzing the behavior of laboratory animals [50]. The Multimodal Multisensor Activity Annotation Tool (MMAAT) offers similar functionalities for multichannel data streams from multiple sensors, like depth channels from 3D cameras and accelerometers from wrist-worn devices [51]. The VGG Image Annotator (VIA) (https://www.robots.ox.ac.uk/~vgg/software/via/ (accessed on 12 December 2024)) is a stand-alone tool that enables manual annotation of images, audio, and video data in a web browser [52]. The Computer Vision Annotation Tool (CVAT) is an open-source system for interactive image and video annotation (https://github.com/opencv/cvat (accessed on 12 December 2024)). It integrates functionalities for scaling video annotation, like automatic pre-annotation based on computer vision models and keyframe-based interpolation of manual annotations, in an easily deployable online platform for large-scale projects. A general overview of interaction methods for video content was presented by Schoeffmann et al. [53].

### 2.3. Methods for Attention-Aware Interfaces

Human gaze can be considered a proxy for human visual attention and thus can enhance gaze-based multimodal interaction [54]. We provide a brief overview of such real-time interactive systems because they can benefit from our presented approach for interactive annotation of mobile eye tracking data. Related work includes approaches for building user interfaces that are aware of the current context or situation [55], including conversational interfaces [56]. For instance, Bulling et al. [57] presented an approach for inferring high-level contextual cues from eye movements to facilitate behavioral monitoring and life-logging. Similarly, Steil and Bulling [58] used topic modeling to detect everyday activities from eye movements in an unsupervised fashion. In a later work, the authors presented an approach for visual attention forecasting in mobile interaction settings, which takes the visual scene and device usage data as additional inputs [59]. Toyama et al. [60] implemented a Museum Guide that uses SIFT (scale-invariant feature transform) features [61] with the nearest neighbor algorithm and a threshold-based event detection to recognize user attention to one of 12 exhibits. They extended their approach to detecting read texts and fixated faces with the goal of building artificial episodic memories to support dementia patients [62]. Other approaches combine visual features of a scene with gaze information to detect actions recently performed by a user [63,64,65,66]. Prasov and Chai [67] developed a system that combines speech and passive gaze input to enhance reference resolution in conversational interfaces. Baur et al. [68] implemented NovA, a system for analyzing and interpreting social signals in multimodal interactions with a conversational agent, which integrates eye tracking technology. Thomason et al. [69] developed a gaze-based dialog system that enables the grounding of word meanings in multimodal robot perception. Uppal et al. [5] presented a method for segmenting the fixated object using an end-to-end computer vision model. Chang et al. [70] developed the MemX system that detects human visual attention based on mobile eye tracking and automatically extracts important video sequences that can be used for, e.g., lifelogging. Meyer et al. [71] proposed to use head and eye movement in combination with other sensor data to recognize human activities for building context-aware smart glasses.

## 3. Materials and Methods

We implement the eyeNotate system, a web-based tool for fixation-to-AOI mapping, and evaluate its usability, effectiveness, and efficiency in a small expert case study (n = 3). Further, we conduct a post hoc experiment to assess the performance of the underlying machine learning models in automatically annotating long recordings from head-mounted eye trackers. In the following, we present the details about the implementation of eyeNotate and the methodology used for evaluating it.

### 3.1. The eyeNotate Annotation Tool

**Figure 1:**
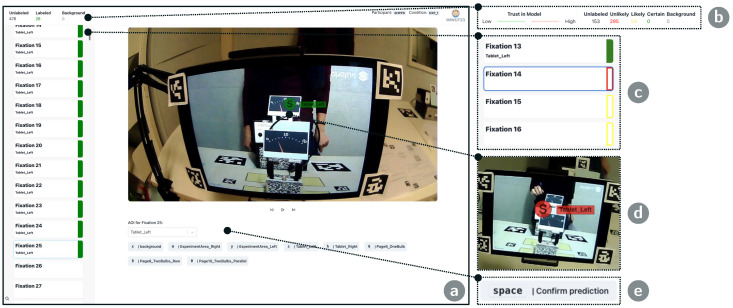
(**a**) Screenshot of the user interface of our baseline annotation tool and (**b**–**e**) an overview of the IML-support features. It extends the baseline by (**b**) a status bar indicating the number of AOI suggestions grouped by model certainty and a trust-level slider for adjusting certainty intervals, (**c**) indicators for AOI suggestions in the fixation list, (**d**) adjusted fixation overlays for the video, and (**e**) an option to confirm AOI suggestions.

We implement eyeNotate, a web-based tool for fixation-to-AOI mapping, an essential data processing step in research based on mobile eye trackers. Our tool allows practitioners to annotate recordings manually fixation-wise, reflecting the current state of the art (baseline). We designed the user interface to enable efficient navigation through videos based on fixation events aligned to common video-editing interfaces. Further, we integrate an IML component that can provide AOI label suggestions for fixations and learn from user feedback, i.e., when they accept or reject/correct suggestions, based on a few-shot image classification model (IML-support). User annotations and model-based suggestions are stored in a database. Figure 1 shows the basic user interface and an overview of the IML-support features.

#### 3.1.1. Baseline Annotation Tool

The baseline tool offers a video-editing-like interface for fixation-wise data annotation (see Figure 1a). It includes three main elements: A top bar displays information on the selected recording and the annotation progress, a list on the left shows all fixations and their annotation state, and a video view on the right with a fixation overlay and buttons for manual annotation. Selecting a fixation from the list causes the video view to show the respective image frame with a circular overlay at the fixation position, indicating the currently assigned AOI. An AOI can be assigned to the fixation by clicking one of the AOI buttons or pressing the corresponding shortcut on the keyboard. This is visually confirmed by a green badge that appears next to the fixation’s list entry, and the overlay in the video view that turns green and shows the newly assigned AOI label. Navigation through fixations is possible via the arrow keys and on-screen video controls. When consecutive fixations hit the same AOI, they can be annotated simultaneously by selecting multiple fixations from the list using the shift and arrow keys in combination. This is consistent with multi-item selection features in common list views.

#### 3.1.2. Interactive Machine Learning Support

**Figure 2:**
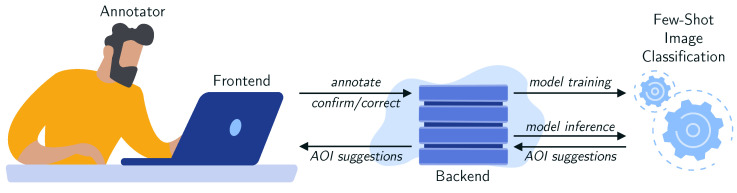
Overview of the architecture of our interactive annotation system, including a web-based user interface (frontend), a backend for managing data storage, and an IML service that enables label suggestions and model retraining for the IML-support version of our tool.

The IML-support version of our tool integrates an IML component based on a few-shot image classification model, which is initialized with a small set of images per AOI. This model generates AOI label suggestions for each fixation by cropping an image patch from the corresponding video frame around the fixation point. Manual annotations and confirmatory or corrective feedback are used to re-train the image classification model, aiming to improve its performance over time. The model training and inference run in parallel to enable flexible and quick adaptations of the model to the target domain. Figure 2 shows a high-level overview of the components of our system and how they interrelate.

##### User Interface

The user interface of the IML-support version is extended to display and interact with model-based label suggestions (see Figure 1b–e). A non-filled badge at a fixation’s list item indicates that a suggestion is available (see Figure 1c). The outline color of the badge encodes the model’s confidence, which is either high (green), medium (yellow), or low (red). The color is also reflected in the fixation overlay in the video view (Figure 1d). Users can set their perceived trust in the model using a slider in the top bar (Figure 1b). Moving the slider towards high trust decreases the confidence thresholds: more suggestions appear in green. Next to the slider, an overview displays the distribution of suggestions across confidence levels. A suggestion can be confirmed or corrected by users. They press the space key to confirm a suggestion for one or multiple selected fixations (Figure 1e). To correct it, they assign another class.

##### Image Classification Model

**Figure 3:**
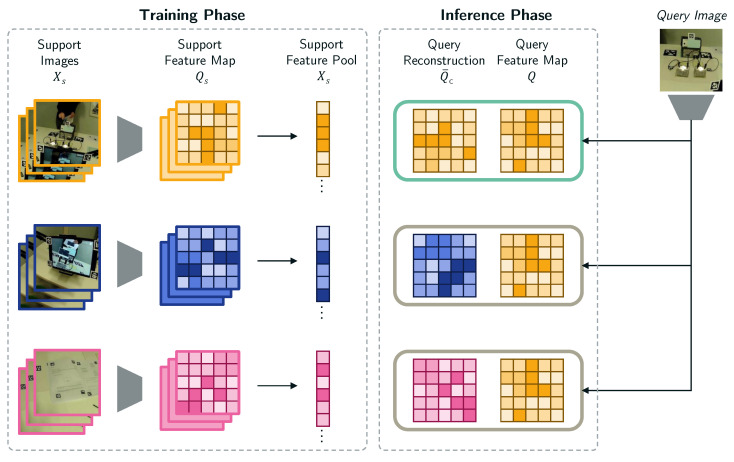
Overview of the FRNet classification workflow for a few-shot classification problem.

The IML-support version adopts a few-shot learning strategy based on the Feature Map Reconstruction Network (FRNet) [72] to generate AOI label suggestions. An overview of the training and inference for this model is illustrated in Figure 3. The FRNet is a convolutional neural network (CNN) architecture that performs classification via a class-agnostic distance function: The image classification task is framed as a reconstruction problem in latent space; i.e., predicting class membership relies on measuring the distance between a query point and reference points in latent space representing our target classes (i.e., AOIs). For any query image *x*, the convolutional block of the network outputs a feature map $Q\in R^{r\times d}$, where *r* is the spatial resolution (h×w) and *d* is the number of channels. The network is trained in an N-shot-K-way manner to learn support feature maps $S_{k}\in R^{Nr\times d}$ for each AOI class k∈K from a pool of *N* training images per class. During inference, the model aims to reconstruct the best-fit query feature map $Q_{k}$ for each class category as a weighted sum of rows of $S_{k}$ such that $WS_{k}\approx Q_{k}$, where *W* is the model weights optimized during model training. By examining the negative reconstruction error, which represents the disparity between the original feature map *Q* and each AOI-wise reconstructed feature map $Q_{k}$, FRNet assigns a class score. Smaller reconstruction errors indicate a higher likelihood that the query image belongs to the same class as the support features. We train our classification model using n = 10 images and for K = 7 AOIs (initial labeled data pool). Following Wertheimer et al. [72], we combine the classification loss with an auxiliary loss $L_{aux}$ that optimizes support features from different classes to span the latent space to train FRNet:

(1) $$L_{aux}=\sum_{i\in K}\sum_{j\in K,j\neq i}{∥S_{i}S_{j}^{T}∥}^{2}$$

The annotation tool uses this pre-trained FRNet model to infer AOI labels for each fixation in the selected dataset. Label suggestions are displayed if the threshold exceeds a minimum confidence value (0.4) that the user can adjust through the trust-level slider. Manual annotations and confirmed or corrected AOI labels are added to the labeled data pool. For every 10 new samples, a model re-training is started in the background. The model weights used for inference are updated upon completion. The models are trained for 30 epochs at each iteration with weights initialized from the previous steps. On an NVIDIA RTX 3080 GPU (24GB), the model training takes 2–4 s per epoch.

### 3.2. Evaluation

We evaluate our approach in two ways: we conduct a small case study with n = 3 trained annotators to quantitatively and qualitatively compare the baseline version of our tool with the IML-support version. Annotators have been asked to annotate a small portion of around 2% of an existing dataset with ground-truth annotations. In a post hoc experiment, we assess the performance of three machine learning models in automatically annotating the remaining part of the dataset. In the following, we describe the use case and the corresponding dataset. Then, we provide details about the case study and the post hoc machine learning experiment.

#### 3.2.1. Use Case and Dataset from Educational Research

The evaluation focuses on educational research as an important eye tracking use case. Most digital and analog learning environments are based on visual information. Hence, gaze behavior is an important observable cue allowing researchers to gain insights into learning processes. Jarodzka et al. [73] specify three main research aims for using eye tracking in educational sciences: The first aim is the improvement of instructional designs by investigating the waste of cognitive resources on ineffective instructional material (see, e.g., Malone et al. [74]). Second, eye tracking can be used to investigate visual expertise leading to superior performance (see, e.g., Reingold and Sheridan [75]). Third, eye tracking can be used to model learners’ eye movements to promote visual expertise (see, e.g., Jarodzka et al. [76]). Some further educational studies also used eye tracking to investigate learners’ gaze behavior in testing situations before and after learning phases (see, e.g., Thees et al. [77]). Recent mobile eye tracking devices are convenient to wear and enable learners to move freely and naturally in dynamic and interactive real-world learning environments, e.g., classrooms or science laboratories [78,79]. This is especially beneficial for eye tracking recordings with children, as they can easily be distracted by intrusive measurements and have difficulties sitting still for long periods of time.

The case study (n = 3) and machine learning experiment described below use recordings from an existing mobile eye tracking dataset (n = 48). It was recorded and annotated at Saarland University. The goal was to investigate the impact of AR-support in a lab work-based learning scenario about electrical circuits on learning outcomes and learning processes of elementary school children (pre-registered at Open Science Framework: https://osf.io/gwhu5; accessed on 12 December 2024).

Tablet-based AR was used to visualize measured values of current in different electric circuits in real time during several experiment and observation tasks. The tablet-based AR condition was compared to a condition in which a separate tablet presented the same values without using AR. The data to be annotated in the current case study originates from a single individual (child) who was assigned to the separate tablet condition. All children wore a Pupil Core head-mounted eye tracker for children [29]. The lab work started after a short introduction and the calibration of the mobile eye tracker through physical markers. The Pupil Capture tool was used to record eye tracking data and a video from the world camera.

The experiments investigated whether children would benefit from AR-based information displays when learning scientific laws on current in series and parallel circuits. In the first experiment, children built a simple electrical circuit with one bulb. While the current at the power supply was manipulated, the children answered questions on the bulb’s brightness and current measurements. After building up a series circuit with two bulbs for the second experiment, the children again observed the current and brightness of bulbs while the current at the power supply changed and answered some questions. Subsequently, the children were asked to compare the brightness and current of the simple circuit they built for the first experiment and the series circuit. The children also carried out a third experiment on parallel circuits, which is not part of the present study. For the current case study, the comparison process within the simple circuit (experiment phase 1 → exp_1) and the comparison process between the simple and the series circuit (experiment phase 2 → exp_2) are examined. An overview of the considered AOIs can be found in Table 1.

**Figure 4:**
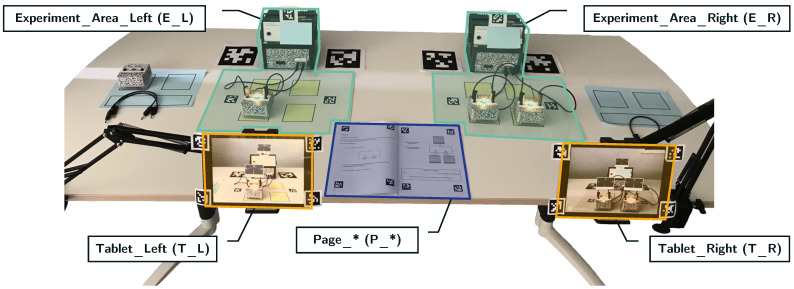
Overview of the experiment setup illustrating considered AOIs.

Figure 4 shows an overview of the experiment scene with overlays for each AOI. Experiment phase 1 includes five AOIs of the simple circuit setup with one bulb placed on the left side of an experimentation table: left tablet with measurement values (Tablet_Left → T_L), left voltage source and electric components (Experiment_Area_Left → E_L), and a double page in a workbook (Page6_OneBulb → P_6). Experiment phase 2 includes additional AOIs of a series circuit placed on the right side of the same table: right tablet with measurement values (Tablet_Right → T_R), right voltage source and electric components (Experiment_Area_Right → E_R), and another double page in a workbook (Page8_TwoBulbs_Row → P_8). The voltage source and electric components’ AOIs per side were merged into a single AOI for analysis. A third double page for phase three was sometimes visible as the children scrolled through the workbook (Page10_TwoBulbs_Parallel → P_10). This results in a total of seven AOIs: three for experiment phase 1, three for experiment phase 2, and one additional for the workbook pages of phase 3. However, the AOIs could have also been visible when not intended because the scene was set up completely, and the children might have looked at non-relevant AOIs. Nevertheless, fixations on these AOIs have been annotated. It is important to note that the tablets, experiment areas, and workbook AOIs have similar appearances, which relates to challenge III outlined in Barz and Sonntag [38].

Following the completion of the data collection, the Pupil Player tool was used for detecting fixation events and annotating the eye tracking data fixation-wise: it offers an option to jump between successive fixations and supports hotkey-based annotation. All recordings have been annotated by four student assistants employed by Saarland University. They received intensive training before the annotation took place. The manual annotation of the full dataset took several days, which led to fatigue, frustration, and, eventually, inadvertent errors in the annotations that were difficult to fix. We recruited three of these student assistants for the present expert study; the fourth did not reply to our invitation.

#### 3.2.2. Case Study

We invited n = 3 trained annotators for evaluating the baseline and IML-support versions of eyeNotate. We measured the perceived usability, annotation validity and reliability, and the efficiency of the annotation process during an annotation task (within-subjects design). Further, we conducted a semi-structured interview to understand how the IML-support version was used and how that might impact the efficiency and validity of the annotation process. For this case study, we focused on the use case of educational research and the existing dataset described above. Next, we provide details about the experiment procedure, the task given to our participants, the specific metrics used for evaluations, and the limitations of this case study.

##### Procedure

We conducted the user study online via video calls and recorded them for post hoc transcription. First, we introduced the study procedure and obtained a signed informed consent via email. Then, we asked annotators to complete an annotation task with both eyeNotate versions. For each, we showed a short instructional video explaining the features. We allowed participants to familiarize themselves with the tool in a 5-min training phase and ask clarification questions. Subsequently, participants performed an annotation task and completed the system usability scale (SUS) questionnaire [80]. Two participants started with the baseline version, one with the IML-support version of the tool. After both annotation tasks were completed, we conducted a semi-structured interview to retrieve further qualitative feedback on our tools, particularly for the distinct features of the IML-support version. The interview guide is provided in Appendix A. The study took around one hour, for which each participant received a EUR 10 compensation payment.

##### Annotation Task

We asked participants to annotate 870 fixations from the dataset described above with ground-truth annotations. This corresponds to around 2% of all samples from the dataset. To reduce the workload in our study, we constrained the annotation task to data from a single child and two experiment phases (exp_1: 646 fixations; exp_2: 224 fixations). In our study, fixations could be mapped to one of seven AOIs or a background class (see Figure 1). The task ended when the participant annotated all fixations. For the IML-support version, the participants could stop early if all fixations had highly confident (“green”) label suggestions, while the confidence level depends on the trust-level slider.

##### Metrics

We measure the perceived usability, annotation validity and reliability, and efficiency during the annotation task to assess the two annotation tool versions. We expect the IML-support version to be more efficient than the baseline, with the perceived usability and annotation validity and reliability remaining stable.

- **Validity and Reliability:** We measure the validity of the participants’ annotations for each tool version. We report their accuracy in mapping fixations to AOIs compared to the ground-truth annotations from the dataset used in this study. Further, we assess the reliability as the level of agreement among all participants for each version of our tool by calculating Fleiss’s κ [81]. We consider both measures to be control variables: we expect to observe a high accuracy for both versions of the tool (≥95%) and an almost perfect inter-rater agreement (κ>0.8) [82].
- **Efficiency:** We measure the time required for completing the annotation tasks in seconds (task completion time) for each tool version. We expect the IML-support version to be more efficient than the baseline, according to findings in prior research, i.e., that the availability of label suggestions leads to easier and faster decision-making [23].
- **Usability:** We assess the usability of both versions of our annotation tool using the system usability scale (SUS) questionnaire [80]. Scores can range between 0 and 100, with high scores indicating better usability. We interpret the SUS scores according to the adjective rating by Bangor et al. [83]. We consider this a control variable; i.e., we do not expect a difference in perceived usability between the two versions of our tool, but we expect a high SUS score for both versions. Further, we conduct a semi-structured interview (SSI) to gain further qualitative insights about our annotation tool and specific IML features. The transcribed interview was analyzed using a reflective thematic analysis [84].

##### Limitations

One limitation of this case study is the small number of three participants. While we expect to gain important insights into the effectiveness, efficiency and usability of our interactive machine learning tool eyeNotate, these results are not generalizable. Further investigations will be required in the future, covering additional use cases, i.e., not restricted to educational science, and additional users and user groups, e.g., lay users that were not previously trained for the annotation task.

#### 3.2.3. Post Hoc Machine Learning Experiment

**Figure 5:**
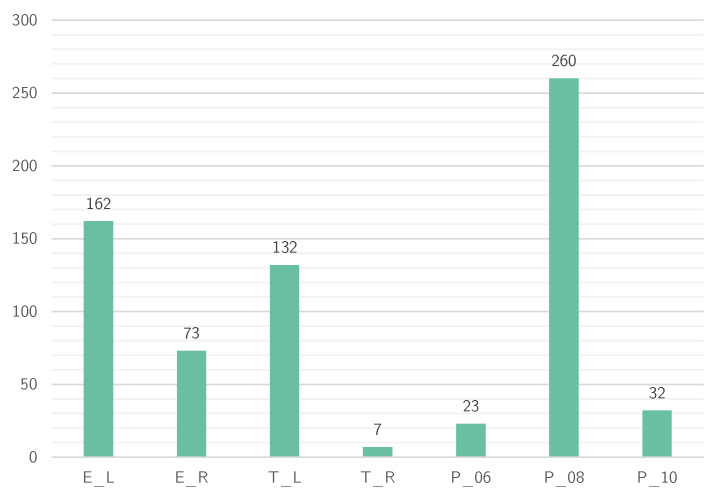
Class distribution on the training set for the post hoc machine learning experiments. *Tablet_Right (T_R)* has the lowest and *Page8_TwoBulbs_Row (P_08)* the highest number of samples.

In a post hoc experiment, we assess the performance of three machine learning models in automatically annotating the part of the dataset that was not annotated during our study; i.e., all test data remains unseen. This includes around 230k fixations from 47 individuals. The automatic fixation-to-AOI mapping includes all seven classes from our experiment, plus a background (BG) class. However, the models are not trained to directly classify the background class. The background class *BG* is assigned if the probability is lower than a threshold $t_{BG}=0.4$. This means fixations are assigned to one of the seven AOIs if the probability for this classification is greater than or equal to $t_{BG}$. The three considered models include the few-shot learning model (FRNet) [72] that was used in our IML-support version; ResNet50 (ResNet) [30], a well-established foundation model for image classification tasks; and MobileNetV2 (MobileNet) [85], a lightweight architecture model suitable for resource-constrained environments. We consider two data settings for model training: *base* and *final*. For the *base* setting, we use the initial labeled data pool with 10 images per class as the training set, i.e., the 70 images that were used to pre-train the FRNet model for the IML-support version of our tool. For the *final* setting, models are trained using ground-truth labels for the 870 fixations from the annotation task. Figure 5 shows the class distribution for the seven AOIs in the training set. However, by that, we assume that a participant correctly annotates all fixations, which is not exactly true but sufficient for our experiment: the average accuracy of our participants in annotating these 870 fixations was 94.55%. In the *final* setting, we train FRNet in a 100-shot, seven-way manner, upsampling images for classes with less than 100 training images because the model requires an equal number of samples per class (random oversampling). Instead of upsampling, we use *weighted cross-entropy* classification loss to train ResNet and MobileNet, which addresses the class imbalance. As described above, an additional loss with a scaling factor of 0.03 is used to train FRNet. All models are trained for 30 epochs using an SGD optimizer with a learning rate of 0.0001. We report the accuracy and f1 scores of all models.

## 4. Results

In the following, we report the results of our evaluation, including a small case study (n = 3) and a post hoc machine learning experiment to assess the models’ ability to automatically annotate data when additional training data is used.

### 4.1. Results of the Case Study

We present the results for each tool version, i.e., the baseline and IML-support versions. In some cases, we report the individual values per participant because we only considered three trained annotators for our case study: A1, B1, and B2. Participants started with the IML-support (A) or the baseline version (B).

#### 4.1.1. Validity and Reliability

We assess the validity of annotations in terms of their accuracy compared to the ground truth. We report the mean over all three participants for each version of the annotation tool per phase and combined (see Table 2). For phase *exp_1*, we observe an accuracy of 97.32% for the baseline version and 97.78% for the IML-support version. We observed slightly lower values for phase *exp_2*: the accuracy is 89.58% for the baseline version and 88.24% for the IML-support version. The weighted average over both phases results in an accuracy of 94.76% for the baseline version and 94.55% for the IML-support version. This weighted average considers the unbalanced number of fixations in each phase. We calculate Fleiss κ as a measure for the inter-rater agreement. It is calculated per condition and phase based on the ratings from all three participants. Table 2 shows agreement values that range between 0.919 to 0.963 (almost perfect). On average, we observed no deviations in validity or reliability comparing the two versions of our annotation tool.

#### 4.1.2. Efficiency

We analyze the time required for completing the annotation task per tool and user. Overall, the slowest participant was A1, who completed the tasks for the baseline condition in 1999 s and 2095 s for the IML-support condition. Participant B1 was faster, with 1189 s for the baseline condition and 1251 s for the IML-support condition. With 980 s for the baseline condition and 966 s for the IML-support condition, participant B2 was the fastest annotator. While the individual differences in the task completion times are large, we found only small differences in the completion times between the two conditions. On average, our participants required 1389 s to complete the tasks for the baseline condition and 3.44% longer (1437 s) for the IML-support condition. The high rater agreement indicates that there is no relation between task completion time and the validity of the generated annotations.

**Figure 6:**
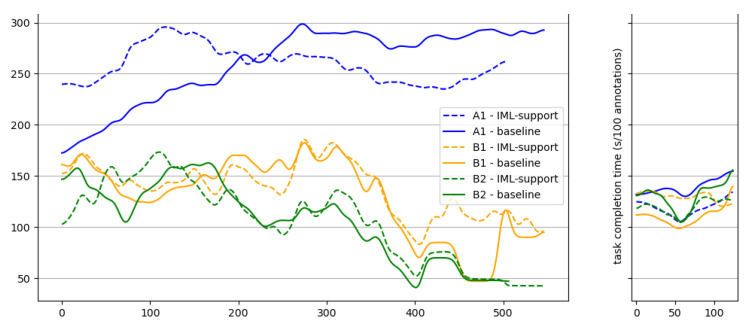
The task completion time per experiment phase, participant, and version of the tool over time.

We also investigate whether the task completion time changes over time. We plot the average task completion time for chunks of 100 samples against time in Figure 6. Hereby, the x-axis determines how many samples have been annotated so far, and the task completion time displays the time that was required for annotating the next chunk of 100 fixations (moving window average with window-size 100 and step-size 1). The diagram shows that differences in task annotation time between users originate from annotating fixations from experiment phase 1 (exp_1). A1, who was the slowest overall, has consistently higher task completion times per 100-fixation chunk than the two other participants, B1 and B2. A1 requires around 250 s/chunk when using the IML-support version. When using the baseline version, A1 is faster in the beginning (lower than 200 s/chunk), but task completion time increases to almost 300 s/chunk towards the end. B1 and B2 need around 150 s/chunk in the beginning. Towards the end, task completion times improve for both participants. B1 takes around 100 s/chunk in the end, B2 around 50 s/chunk. We cannot observe differences between the IML-support and the baseline versions of our tool. The task completion times for experiment phase two lie between 100 s and 150 s per chunk for all participants and versions of eyeNotate.

In an additional analysis, we assessed differences in annotation times between different classes. Table 3 shows the mean number of annotations per class (overall), and the mean task completion time in seconds per annotation for the two versions of eyeNotate. The table shows that users required the most time for annotating fixations belonging to the classes T_R, P_10, and BG, with T_R and P_10 having the least annotations overall, independent of the version of eyeNotate. We observed the fastest annotation times for E_R, P_6, and P_8. The time required for annotating E_L and T_L depends on the tool used. In both cases, the annotation times were lower when using the baseline version compared to the IML-support version.

#### 4.1.3. Usability

We measured perceived usability using the SUS questionnaire. The baseline version is consistently rated as “excellent” with values ranging from 87.5 to 95 (91.6 on average). For the IML-support condition, we observed a high variance in SUS scores: the ratings range from 50 for B1 (“poor”) to 67.5 for B2 (“OK”) to 97.5 for A1 (“excellent”), averaging to 71.6. The reflexive thematic analysis of the SSI revealed two themes: (a) the tool’s design facilitates the annotation of mobile eye tracking data, and (b) the constrained model performance limits IML-based benefits. Details are provided in the discussion section below.

### 4.2. Results of the Machine Learning Experiment

Table 4 reports the accuracy for each model and training setting. FRNet outperforms MobileNet and ResNet: it achieves an accuracy of 57.57% in the *base* setting and 58.78% in the *final* setting, which is 6.64% and 7.39% better than the second-best models, respectively. The model performs marginally better when taking the annotations of our participants into account for training in the *final* setting (+1.21%). MobileNet ranks second for the *base* setting with an accuracy of 50.93%. The accuracy slightly decreases to 49.28% for the *final* setting. ResNet performs worst for the *base* setting with 39.60% and benefits most from using more training samples in the *final* setting. The accuracy increases by 11.78% to 51.39%, now slightly outperforming MobileNet.

Table 5 reports the class-wise and averaged f1 scores for each model and training setting. In both training settings, FRNet performs best in terms of the macro and weighted average of the f1 score. The best performance is achieved in the *final* setting with a macro-average f1 score of 0.455 and a weighted average of 0.593. In the *base* setting, the macro-average is 0.428, and the weighted average is 0.579. MobileNet and ResNet achieve considerably worse average f1 scores in both settings. For the *base* setting, the macro-average is 0.202 for MobileNet and 0.256 for RestNet, the weighted average is 0.460 for MobileNet and 0.409 for ResNet. MobileNet does not benefit from taking more training samples into account in the *final* setting: the macro-average f1 score slightly drops to 0.185, and the weighted average f1 score to 0.445. For ResNet, the macro-average f1 score stays similar, while the weighted average f1 score improves by 0.062 to 0.471. However, this is still 0.122 worse compared to FRNet in the same setting and 0.107 worse than FRNet in the *base* setting. It is noteworthy that the difference between FRNet and the other two models is larger for the macro-average f1 score (difference ≥ 0.172) than for the weighted average f1 score (difference ≥ 0.119). Also, the macro-average f1 score is always clearly worse than the weighted average f1 score, indicating that all models perform better for classes with many samples than for classes with a small number of samples. A class-wise analysis shows that all models perform best for the background class (*BG*) with f1 scores starting from 0.569 for ResNet in the *base* setting and larger than 0.663 for all other conditions. The best performance for the background class was observed for ResNet and FRNet in the *final* setting with an f1 score of 0.681. We only observed a single better f1 score of 0.687 for the tablet class *T_L* for FRNet in the *final* setting. As the background class covers more than half of all samples (137.9k of 230.3k samples), it has a large impact on the weighted average. For MobileNet and ResNet models, we observed low f1 scores of less than 0.5 for all seven classes other than *BG* in both settings. FRNet shows a more balanced performance. In the *base* setting, only four out of eight classes achieve an f1 score below that threshold. Further, for FRNet, we observed the best performance for each class besides *P_10* for which MobileNet was better. In the *final* setting, FRNet improves for all classes besides the experiment area *E_R* (−0.094), which is why five out of eight classes have an f1 score lower than 0.5. Still, the model performs best for all classes besides *P_10*. For *BG*, ResNet performs equally well in this setting. The best f1 scores for FRNet are observed for the background class *BG* and the two tablet classes *T_L* and *T_R*.

**Figure 7:**
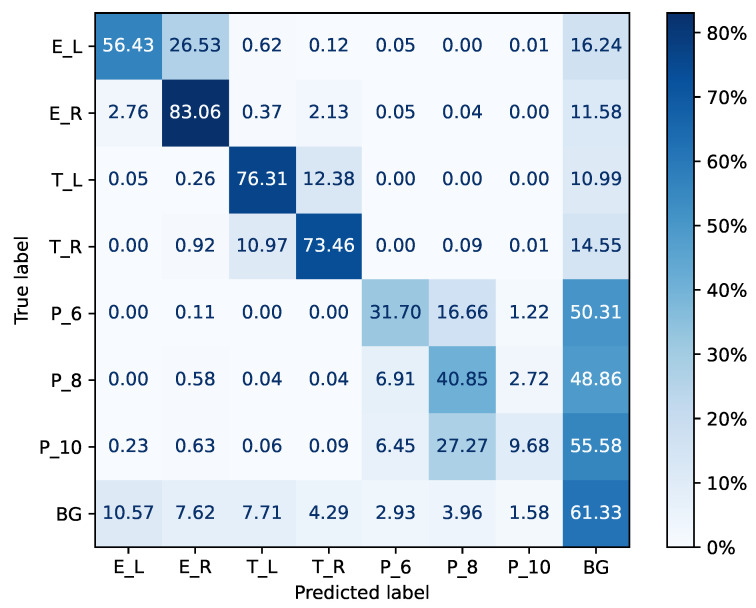
Confusion matrix for the test set for FRNet in *final* setting (normalized over rows).

Figure 7 shows the confusion matrix of the best-performing condition: FRNet in the *final* setting. It is normalized over the true conditions (i.e., over rows): the values on the diagonal correspond to the recall of a respective class. Other values in the same row correspond to false-negative errors and sum up to the miss-rate of that class. For instance, for the background class *BG*, the recall is 61.33%, and the false negatives sum up to a miss rate of 38.66%. The background is often misclassified as one of the experiment area classes (18.19%) or as one of the tablet classes (12%). The confusion matrix shows that classes with similar appearances are frequently confused. This can be observed for the two experiment area AOIs, the two tablet AOIs, and the three workbook AOIs. For instance, for *E_L*, the recall is 56.43%, and, with 26.53%, the majority of the false negatives were classified as *E_R*. The recall of *T_L* is 76.31% while 12.38% of the false negatives were classified as *T_R*. A similar pattern was observed for the workbook AOIs P_*. All AOI classes are frequently misclassified as background. Hereby, the false-negative errors for the experiment area and tablet AOIs range between 10.99% and 16.24%. The three workbook AOIs are affected more severely: the false-negative errors range between 48.86% and 55.58%. This results in a precision of 0.765 for *BG*, which is the best precision among all classes. Precision and recall for all classes are reported in Table 6.

## 5. Discussion

With eyeNotate, we present a tool for annotating mobile eye tracking data. Our goal is to create a tool that allows researchers to more effectively and efficiently annotate recordings from mobile eye trackers while providing a high usability. In the following, we discuss the results of our evaluation, including a case study with three trained annotators and a post hoc machine learning experiment.

### 5.1. Validity and Reliability

The validity of users’ annotations is high and alike for both versions of our annotation tool. We observed an accuracy of 94.76% for the baseline version and 94.55% for the IML-support version (weighted mean). An additional analysis revealed 14 errors (1.6%) in the ground truth. We identified these errors in cases when all three annotators agreed on an AOI that deviated from the ground truth. With a corrected ground truth, accuracy increases to 96.29% for the baseline version and 96.07% for the IML-support version. This suggests we met our goal of achieving an accuracy of at least 95%. Our results further suggest that the *exp_2* was more difficult to annotate because accuracy values consistently dropped for both versions of the tool from more than 97% accuracy to less than 90%, and we observed a higher ratio of ground-truth errors. A reason might be that the second phase included more different AOI classes and a more complex scene. The inter-rater agreement was almost perfect with κ≥0.9 in all cases, i.e., the reliability of annotations from both versions of our tool is high.

### 5.2. Efficiency

On average, task completion times for both tool versions were similar: annotators were 3.44% (48 s) slower when using the IML-support version. Likewise, the difference in task completion times between versions per participant is small. On the other hand, the differences between participants are large. A1 required around 2000 s to solve the task per tool, while B1 and B2 required around 1200 s and below 1000 s, respectively. This is almost twice as fast without compromising accuracy, which indicates that B1 and B2 had a more efficient strategy in using our tools. Analyzing the task completion times over time, we observe that A1 is consistently slower than B1 and B2 with annotation times of 250–300 s/100 annotations. B1 and B2 require only around 150 s/100 annotations. During the study, we observed that all participants used shortcuts for annotation and confirmation, but A1 did not use the multi-select feature, which could explain the high difference to B1 and B2 in terms of task completion time. Another indicator for the high effectiveness of the multi-select feature is that B1 and B2 had the lowest task completion times (50–100 s/100 annotations) at the end of *exp_1*, which includes many consecutive occurrences of P_6 and P_8 (see also the low class-wise annotation times in Table 3). Overall, given the 870 fixations in the annotation task, our eyeNotate achieves a worst-case annotation rate of 2.41 s/fixation for user A1 when using the IML-support version and a best-case annotation rate of 1.11 s/fixation for user B2 when using the IML-support version. This means, using an automatic annotation method to map the remaining 230k fixations in the full dataset, there is a time-saving potential between 70 and 150 h for this use case.

However, we could not confirm our hypothesis that providing label suggestions would accelerate the labeling process. This is likely because all annotators tended to manually check and confirm label suggestions in the IML-support version (cf. Section 5.3). We observed corresponding annotation behavior during the study, and theme (b) of our SSI analysis concerning the constrained model performance confirms this: annotators did not trust the model sufficiently and felt highly responsible for performing the job well. Hence, they did not benefit from automatic label suggestions as found in Desmond et al. [23]. The differences in interaction design between the baseline and the IML-support version of our tool seemingly played no role in this context. Our findings from the SSI analysis relating to theme (a) suggest that participants, in principle, liked the interaction design of the IML-support version, but due to the low model performance, these features were not effective. Our findings suggest that future investigations should include more effective computer vision models that can better cope with the challenges of mobile eye tracking data like differentiating classes with similar appearance. This could, for instance, be achieved using a classification model that takes the position of a fixated object into account [86] or by tracking objects once they have been annotated once using 3D scene reconstruction and object tracking algorithms [87]. Follow-up work could also investigate how lay users, in contrast to the trained annotators in our case study, perform in the annotation task, following the questions whether lay users could achieve the same validity as trained annotators and whether lay users would benefit more from label suggestions in terms of efficiency.

### 5.3. Usability

The usability of our tool’s baseline version was consistently rated as excellent: the basic features and general interaction design of our annotation tool were perceived very positively, which is supported by theme (a) of our thematic analysis concerning the tool’s interaction design: “the tool’s design facilitates the annotation of mobile eye tracking data.” However, B1 and B2 rated the IML-supported version drastically lower, which contradicts our assumption that both tools achieve a similar usability rating. Looking into individual SUS items, B1 and B2 majorly penalized an increased inconsistency of the IML-support version and indicated that it was more cumbersome to use. Both felt less confident using the IML-support version and thought it was less easy to use. Particularly, B1, who rated the usability of the IML-support version as “poor”, reported that the system provided many wrong label suggestions and seemed uncertain in many cases, which caused confusion and deteriorated trust. B1 reports that, as a consequence, they fell back to a manual annotation strategy. B2 and A1 reported similar issues with the model performance despite rating usability higher. We observed that B2 and A1 favored manual annotation, similar to B1. These usability issues can be attributed to the integration of IML-support features and relate to theme (b) of our thematic analysis concerning the constrained model performance: “the constrained model performance limits IML-based benefits.” The two themes, originating from a reflexive thematic analysis of the SSI, are detailed below.

#### 5.3.1. (a) The Tool’s Design Facilitates the Annotation of Mobile Eye Tracking Data

Our case study participants liked our tool’s basic functionality and interaction design. In particular, they highlighted the clean design that allowed them to focus on the annotation task throughout the study. They reported high usability and learnability. Quick reaction times and visual feedback were highly appreciated. Particularly, the video overlay immediately displaying updates after manual annotation or confirmation was considered very helpful because they had to check the video frame to decide on the AOI class anyway. All participants reported a high perceived performance due to the clean, focused interaction design and the ability to use shortcuts for navigation and annotation. Also, the multi-select feature for annotation and confirmation seems to impact annotation efficiency positively. The video playback function was not used by our participants but might have supported understanding the video-editing-like interface metaphor. Upon asking them, participants reported they understood the trust-level slider but did not use it often, although it was considered useful. High-certainty suggestions (green highlight) were also considered helpful. However, certain but wrong label suggestions were frustrating as they could lead to wrong confirmations. Also, the red color of uncertain suggestions was reported to interrupt the interaction flow in the case the predictions were correct. In summary, color-coding of the model certainty for label suggestions might cause frustration in the case of certain but wrong predictions and can interrupt the interaction flow in the case of uncertain but correct predictions. An implication could be to restrict label suggestions to highly certain suggestions. Our participants suggested two interesting features that will be considered in future versions of our tool. They proposed a feature that enables jumping to non-annotated fixations or uncertain suggestions. Further, they proposed a feature to batch-accept all certain predictions, which would be dependent on the state of the trust-level slider and could be restricted to classes with good classification performance.

#### 5.3.2. (b) The Constrained Model Performance Limits IML-Based Benefits

All participants reported a perceived model performance of 30–40% accuracy, although the true value is higher (62%). This indicates that our participants had low trust in the underlying model generating the AOI label suggestions and could explain why they checked all suggestions manually. This is also in line with their reports on problems with certainty-based color coding. All participants specified that the model suffered from a left/right weakness: Some AOIs with the same appearance were present on the left and right sides of the experiment scene, but the model could not properly differentiate between them. We intentionally investigated this challenge by including experiment phase 2. One example is *T_L* and *T_R*, referring to two instances of the same tablet mounted on the left or right side of the experiment scene. This is evident in the confusion matrix for FRNet in Figure 7: *T_L* is wrongly classified as *T_R* in 12.38% of the cases. The false-negative errors concerning all other classes besides *BG* sum up to 0.31%. We observe similar problems for the *experiment area* and *workbook page* AOIs. If objects look very much alike, our IML-support version has limitations. Addressing the left/right weakness is essential because AOIs with similar appearances are common. Future research should investigate whether object-tracking or position-aware models can help to address this challenge. Another option can be found in meta-models that iteratively learn for which classes a model performs well and activate suggestions for those only.

### 5.4. Post Hoc ML Experiment

We observed the best average f1 scores and accuracy scores when using the FRNet model architecture in the final setting, i.e., when using the 870 annotated fixations for training (see Table 4 and Table 5). However, using more training data for the FRNet model only slightly increases the performance, e.g., +1.21% in accuracy and +0.015 concerning the weighted average f1 score. With +11.78% for accuracy and +0.062 for the weighted f1 score, ResNet showed the greatest improvement when more training samples were added. MobileNet performs slightly worse for all metrics. However, the results show that the models are not good enough for most applications such as automatic or semi-automatic annotation with humans-in-the-loop. This is in line with the user’s feedback from the SSI as summarized in theme (b).

The best f1 score of 0.687 was observed for the *T_L* class for the FRNet model in the *final* setting, followed by an f1 score of 0.681 for the *BG* class. The precision is highest for *BG* with 0.765 (see Table 6), so labeling support only for the *BG* class could have been effective. Since almost 60% of all labels belong to this class, this could already save a lot of time without raising usability issues like the ones mentioned in theme (b). The high ratio of *BG* samples in the test set also means that summary statistics like accuracy and the weighted f1 score are biased through the relatively high performance for this class. This is visible in the large deviation between the weighted and macro-average f1 scores for all models. Overall, FRNet shows the most balanced performance across all classes: it performs best for all classes besides *P_10*. This also explains the greater relative difference in the macro-average values and the weighted average values for f1 for MobileNet and ResNet.

The confusion matrix in Figure 7 shows the strengths and weaknesses of the FRNet model (final) on the class level in more detail. As counts are normalized over the true condition, i.e., over rows, the diagonal shows the recall scores for the true condition or class of that row, while the remaining values of that row sum up to the corresponding miss rate. For *BG*, we observed a recall of 61.33% with a precision of 76.53%. This means that, when limiting suggestions to the *BG* class, labels for more than one-third of all instances (61.33% of 59.88% of all 230.3k instances) could have been provided, of which around three-quarters would have been correct. Still, one-quarter would have been wrong. So, limiting suggestions to *BG* alone would likely not solve the usability issues mentioned in theme (b). These scores were observed for the default setting when *BG* is assigned if the model’s classification probability for an AOI class is lower than $t_{BG}=0.4$. Lowering $t_{BG}$ would increase the precision for the *BG* class but at the cost of a lower recall. Likewise, increasing the threshold for assigning one of the seven AOI classes, we call it $t_{AOI}$, would increase the precision for these classes. Eventually, a class-specific batch-accept feature for accepting label suggestions for a certain class with manually tuned $t_{BG}$ and $t_{AOI}$ could be useful. The user should be able to configure the probability threshold $t_{BG}$ and the classification thresholds $t_{AOI}$ for each class, which would allow annotators to accept labels based on their own experiences of how the model performs per class. However, most f1 scores and all precision scores for AOI classes are lower than the scores for the *BG* class (see Table 6), which indicates that tuning the thresholds for a batch-accept feature might be difficult. We conduct and report on a follow-up experiment that investigates how changes in $t_{BG}$ and $t_{AOI}$ affect the classification performance and relate to the number of fixations without a label suggestion. By that, we aim to estimate the potential of a class-wise batch-accept feature.

The confusion matrix also indicates that a reason for the low f1 scores is the similar appearance of the AOI classes, including the two experiment areas E_*, the two tablets T_*, and the three workbook pages P_*. These three groups can be clearly identified along the diagonal as three squares based on the high number of false-negative errors within each group. Further, it shows that many AOI classes are frequently misclassified as belonging to the background class *BG*, particularly the three workbook AOIs. Confusion of AOI classes with the *BG* class could be reduced by increasing the classification threshold $t_{AOI}$. This could be realized, e.g., through a class-based trust-level slider. Confusion of similar-looking AOI classes can only be solved by using more suitable approaches like multi-object tracking; i.e., once an AOI was manually labeled or confirmed by a user, the system could track this instance to reveal wrong classifications or auto-confirm true classification, or graph neural networks that consider the spatial location of an object for classification [86]. An option to increase the utility of the FRNet model would be to provide label suggestions at a higher semantic level. For instance, eyeNotate could identify all tablets and ask the user which instances belong to the left (*T_L*) or right (*T_R*) class. Similarly, this could be performed for the two experiment areas and the three workbook pages. Classification performance would likely be higher for this four-class problem because it is a less complex classification problem. We investigate this aspect in another follow-up experiment. Further, a two-level decision task (left vs. right) or three-level decision task in the case of the workbook pages is less difficult for users than the eight-level decision task, which includes all AOIs and the separate background class.

Next, we report on the the two mentioned follow-up experiments: one for estimating the utility of a class-wise batch-accept feature and one for investigating how the model would perform for the four-class classification problem.

#### 5.4.1. Estimating the Utility of a Class-Wise Batch-Accept Feature

To estimate the utility of a class-wise batch-accept feature, we investigate the impact of adjusting the classification thresholds $t_{BG}$ and $t_{AOI}$ on the model performance in an additional experiment. In the current setting, eyeNotate suggested *BG* as a label when the probability was below a threshold of $t_{BG}=0.4$ and the highest-ranked AOI class otherwise. In this post hoc experiment, we add a second threshold $t_{AOI}$ that determines the minimum classification probability *p* before we assign an AOI class. The higher the gap between these two thresholds, the higher the number of instances without a label suggestion will be. Hence, there will be a trade-off between the number of instances with a label suggestion and the precision of those.

**Figure 8:**
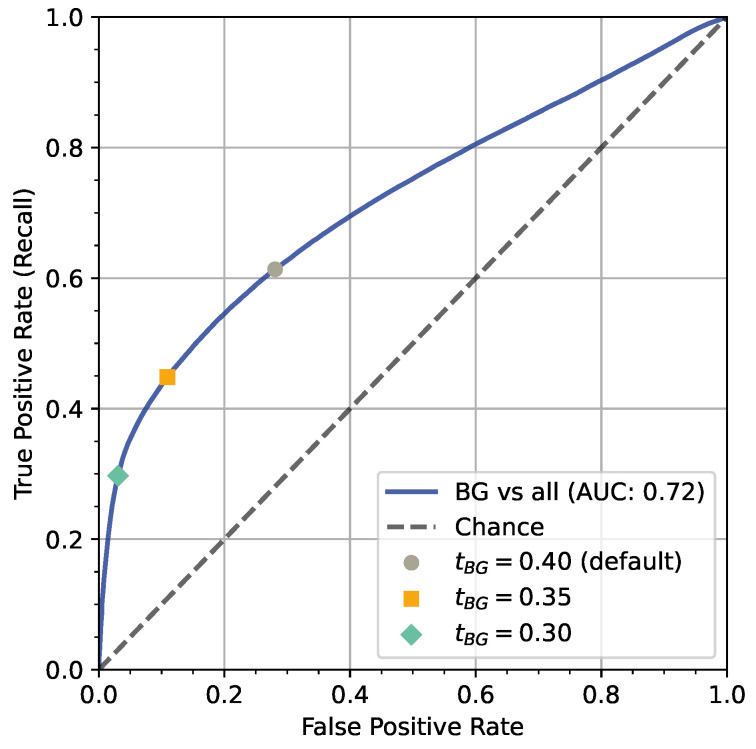
ROC curve for the background class *BG* for the FRNet model in the *final* setting. The decision boundary corresponds to the threshold $t_{BG}=t_{AOI}$.

In the first step, we assess whether the default threshold for classifying the background class $t_{BG}=0.4$ was a good choice. For this, we plot an ROC curve that illustrates the trade-off between the true-positive rate (recall) and the false-positive rate for classifying the *BG* class (versus all other AOI classes) depending on $t_{BG}$ (see Figure 8). Note that in the default setting, $t_{AOI}=t_{BG}$. The ROC curve shows that false-positive rate for $t_{BG}=0.4$ is quite high: 28.07% of non-*BG* instances are wrongly classified as *BG*. Reducing $t_{BG}$ to 0.35 or 0.30 improves the false-positive rate: only 10.92% or 3.06% are wrongly classified as background. The recall would drop to 44.83% and 29.96%, respectively. A recall of 29.96% still corresponds to 17.94% of all samples (41.3k) because 59.88% of all 230.3k samples belong to the *BG* class.

**Figure 9:**
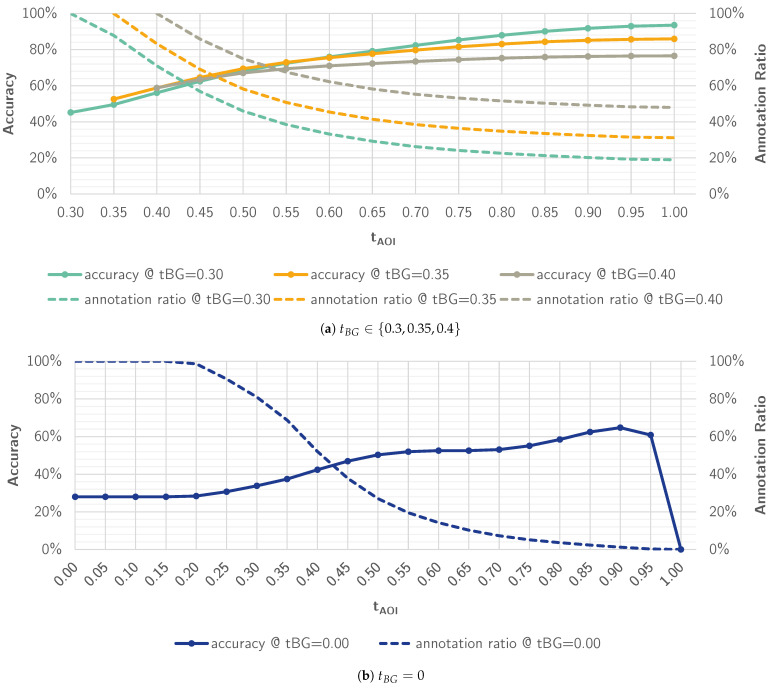
Accuracy and annotation ratio as a function of $t_{AOI}$ for the FRNet model in *final* setting for $t_{BG}\in \left\{0.3,0.35,0.4\right\}$ (**a**) and for $t_{BG}=0$ (**b**).

However, simultaneously reducing $t_{BG}$ and $t_{AOI}$ optimizes the false-positive rate for the background class but will also lead to an increase in false-positive rates for all other classes. Hence, we investigate the impact of increasing $t_{AOI}$ in 5% steps on accuracy with constant $t_{BG}$ for $t_{BG}\in \left\{0.3,0.35,0.4\right\}$. At the same time, we investigate the impact on the number of samples that will not be annotated. The results are presented in Figure 9a. It shows the model accuracy and the annotation ratio, i.e., the portion of samples that received an annotation suggestion, as a function of $t_{AOI}$. Using the default parameters $t_{BG}=t_{AOI}=0.4$, we observe an accuracy of 58.78% as reported in Table 4 for FRNet in the *final* setting. The annotation ratio is 100% because $t_{BG}=t_{AOI}$. For $t_{BG}=t_{AOI}=0.3$, the curve starts with an accuracy of 45.15%. For $t_{BG}=t_{AOI}=0.35$, accuracy starts with 52.58%. In all three cases, the accuracy increases and the annotation ratio decreases with increasing $t_{AOI}$. Setting $t_{AOI}=1$ means, we do not consider annotations for any class besides *BG*. For $t_{BG}=0.4$, the accuracy reaches 76.53% and the annotation ratio 57.96% in this setting. We observe that the lower $t_{BG}$, the lower the accuracy, and the higher the annotation ratio. Consequently, the maximum accuracy is reached for $t_{BG}=0.3$ with 93.54% as well as the minimum annotation ratio of 18.97%. However, for $t_{AOI}=1$, prediction labels would be limited to *BG*. This indicates that a batch-accept feature for *BG* could be effective. For a batch-accept feature that includes other classes than *BG*, $t_{AOI}$ must be smaller than 1. To assess how well the model would perform for AOI classes only, i.e., for all classes besides the background class *BG*, we ran the experiment for $t_{BG}=0$ and $0\leq t_{AOI}\leq 1$. The corresponding diagram is shown in Figure 9b. Up to $t_{AOI}=0.15$, all samples are classified as one of the AOI classes. This means that the minimum model certainty lies between 0.15 and 0.2. With increasing $t_{AOI}$ the accuracy also increases until it reaches its maximum for $t_{AOI}=0.9$ with 64.75%. However, with these parameters, only 1.24% of all samples would be annotated.

Overall, the results of this additional experiment indicate that a batch-accept feature for the background class *BG* could add value to eyeNotate. Since the parameters are optimized over the test set, the results can only serve as an upper bound of the performance. In a realistic scenario, the performance with a human optimizing the parameters would lie below this upper bound, but it would, in theory, be reachable for the considered use case, dataset, and model. However, the results also show that the classifier is not good enough for providing label suggestions for AOI classes, even under the assumption that users could tune the decision thresholds. A reason is likely the high similarity between some of the AOI classes.

#### 5.4.2. Simulating Model Performance in a Four-Class Classification Setting

Another option to increase the utility of eyeNotate using the FRNet model is to treat the classification as a four-class problem, i.e., to only differentiate between the background class *BG* and three further AOI classes: experiment area *E*, tablet *T*, and workbook pages *P*. For our use case, the human annotator would still need to decide whether, e.g., the identified tablet is the left or right version. But this decision is less complex than assigning one out of all eight classes. Also, this investigation can reveal the potential benefit of eyeNotate for other, more simple use cases. Hence, we assess the overall accuracy and the precision, recall, and f1 scores under the assumption that only four target classes exist, i.e., *E*, *T*, *P*, *BG*, using the FRNet model in the *final* setting. For this, we replace the true and predicted class labels with the corresponding summary class; e.g., *E_L* and *E_R* are replaced with *E* before computing scores. The *BG* labels do not change.

In the four-class setting, FRNet achieves an accuracy of 65.30%, which is 6.52% better than in the original eight-class setting. Table 7 shows the corresponding precision, recall, and f1 scores. As expected, the scores for summary classes are better compared to the original classes. For instance, for *E*, we observe an f1 score of 0.524, while the f1 scores for *E_L* and *E_R* are 0.384 and 0.463, respectively. This also holds for *T* and *P*. The results do not change for *BG* because there were no changes concerning the background class. Consequently, the macro-average and weighted average f1 scores are also higher. The macro-average f1 score increases by 0.167 and the weighted average f1 score by 0.063.

In summary, reducing the complexity of the classification problem has a positive effect on all observed scores. However, to enable effective annotation support we will need to further improve the model performance. Promising directions that should be investigated include methods like multi-object tracking and graph neural network models.

## 6. Conclusions

We presented eyeNotate, an interactive annotation tool for mobile eye tracking data based on few-shot image classification. The results of a case study confirmed that eyeNotate effectively enables fixation-to-AOI mapping: users liked the basic functionality and interaction design, and the validity and reliability of users’ annotations were high. However, we observed that providing AOI label suggestions in the IML-support version did not increase the efficiency, likely because of performance issues of the model that led to low trust in the trained annotators. Still, our results suggested that FSL bears great potential for initiating interactive data annotation. Overall, the task completion times were low, with 1.11 s per annotation (best case) to 2.41 s (worst case). Participants identified constrained model performance as the main hindering factor, especially problems with similar-looking AOIs. This limitation was confirmed in our post hoc machine learning experiment. Future research should aim to develop or integrate more sophisticated computer vision methods that can cope with the dynamic and complex nature of mobile eye tracking data, for instance, using multi-object tracking, 3D reconstruction methods [87], and graph neural networks [86]. Further, the presented case study is limited by its small sample size, consisting of only three trained annotators, which restricts the generalizability of our findings. Future studies should include a larger and more diverse participant pool, e.g., involving untrained lay users that may benefit more from interactive machine learning support. This would be an important contribution to investigating the role of humans in interaction with machine learning algorithms [25].

## Figures and Tables

**Table 1:** List of AOIs indicating their (intended) visibility per experiment phase.

AOI	Visibility	
	exp_1	exp_2
Tablet_Left → T_L	✓	✓
Tablet_Right → T_R		✓
Experiment_Area_Left → E_L	✓	✓
Experiment_Area_Right → E_R		✓
Page6_OneBulb → P_6	✓	✓
Page8_TwoBulbs_Row → P_8		✓
Page10_TwoBulbs_Parallel → P_10		

**Table 2:** Annotations’ validity (accuracy) and reliability (Fleiss’s κ) per experiment phase and as a weighted mean.

	Accuracy		Fleiss’ κ	
	Baseline	IML-Support	Baseline	IML-Support
*exp_1*	97.32%	97.78%	0.954	0.963
*exp_2*	89.58%	88.24%	0.941	0.920
*mean*	94.76%	94.55%	0.951	0.952

**Table 3:** Class-wise mean task completion times in terms of seconds per annotation and the mean number of annotations per class.

		Class							
		E_L	E_R	T_L	T_R	P_6	P_8	P_10	BG
Mean number of annotations		270	59	135	10	276	46	2	73
Mean task completion	baseline	1.55	1.24	1.60	3.04	1.44	1.27	2.96	2.59
time [s/annotation]	IML-support	2.13	1.32	1.89	2.10	1.41	1.14	3.50	2.01

**Table 4:** Accuracy for each model and train setting.

Test Samples	*Base* Setting			*Final* Setting		
	MobileNet	ResNet	FRNet	MobileNet	ResNet	FRNet
230.3k	50.93%	39.60%	57.57%	49.28%	51.39%	**58.78%**

**Table 5:** Class-wise f1 scores for each model and train setting.

Class	Test Samples	*Base* Setting			*Final* Setting		
		MobileNet	ResNet	FRNet	MobileNet	ResNet	FRNet
E_L	10,771	0.207	0.180	0.323	0.153	0.224	**0.384**
E_R	7780	0.001	0.481	**0.556**	0.006	0.457	0.463
T_L	26,167	0.077	0.002	0.662	0.057	0.001	**0.687**
T_R	11,407	0.183	0.316	0.570	0.144	0.087	**0.575**
P_6	14,725	0.317	0.256	0.334	0.310	0.320	**0.375**
P_8	10,242	0.003	0.198	0.209	0.014	0.120	**0.329**
P_10	11,392	0.151	0.044	0.093	0.133	**0.153**	0.146
BG	137,852	0.676	0.569	0.678	0.663	**0.681**	**0.681**
Macro Average		0.202	0.256	0.428	0.185	0.255	**0.455**
Weighted Average		0.460	0.409	0.579	0.445	0.471	**0.593**

**Table 6:** Class-wise precision and recall for the FRNet model in the *final* setting.

Class	Precision	Recall
E_L	0.291	0.564
E_R	0.321	0.831
T_L	0.625	0.763
T_R	0.473	0.735
P_6	0.459	0.317
P_8	0.275	0.409
P_10	0.295	0.097
BG	0.765	0.613
Macro Avg.	0.438	0.541
Weighted Avg.	0.633	0.588

**Table 7:** Class-wise precision, recall, and f1 scores for the FRNet model in *final* setting for a reduced set of four target classes.

AOI	# Samples	Precision	Recall	f1 Score
E	18,551	0.380	0.842	0.524
T	37,574	0.661	0.874	0.753
P	36,359	0.598	0.479	0.532
BG	137,852	0.765	0.613	0.681
macro avg		0.601	0.702	0.622
weighted avg		0.691	0.653	0.656

**Figure A1:**
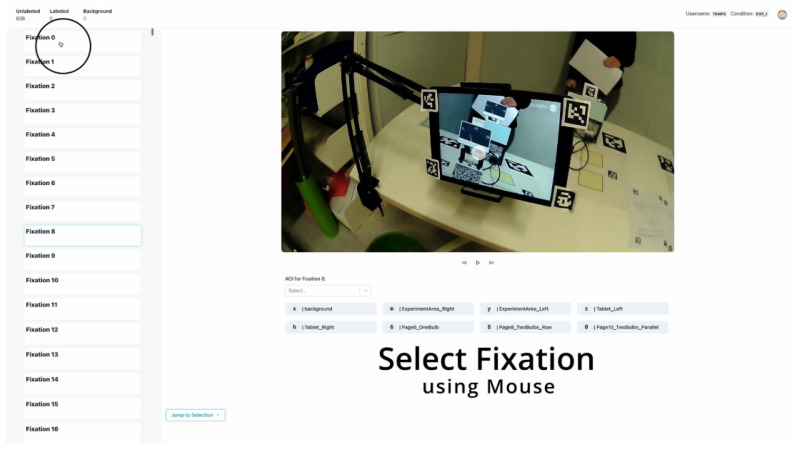
Fixation selection via mouse input.

**Figure A2:**
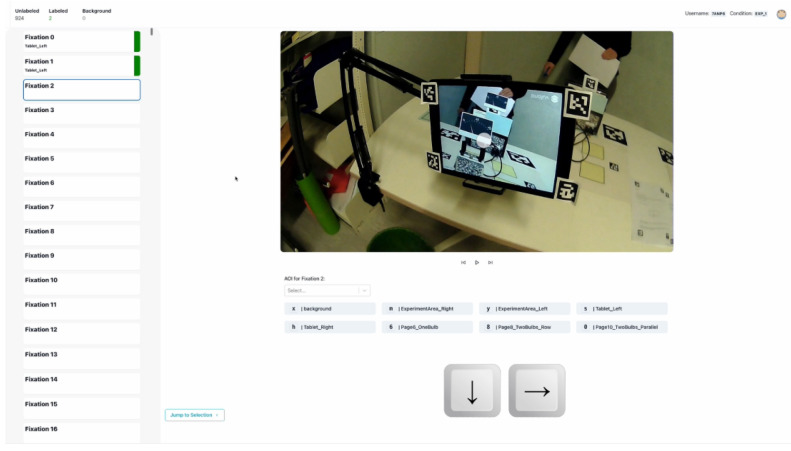
Fixation selection via keyboard input.

**Figure A3:**
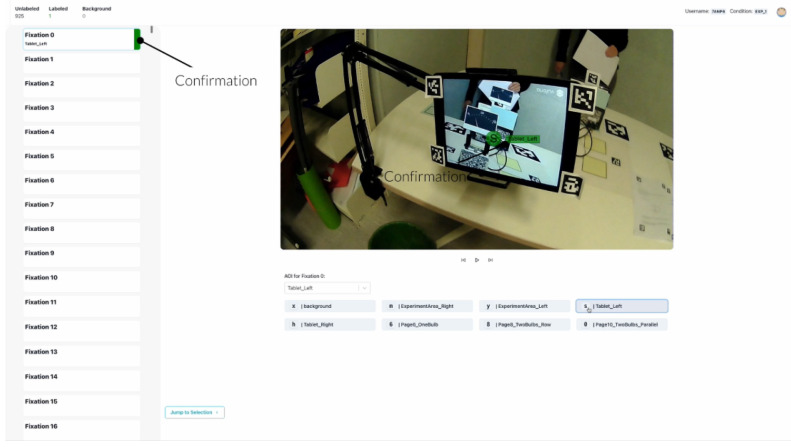
Visual annotation feedback.

**Figure A4:**
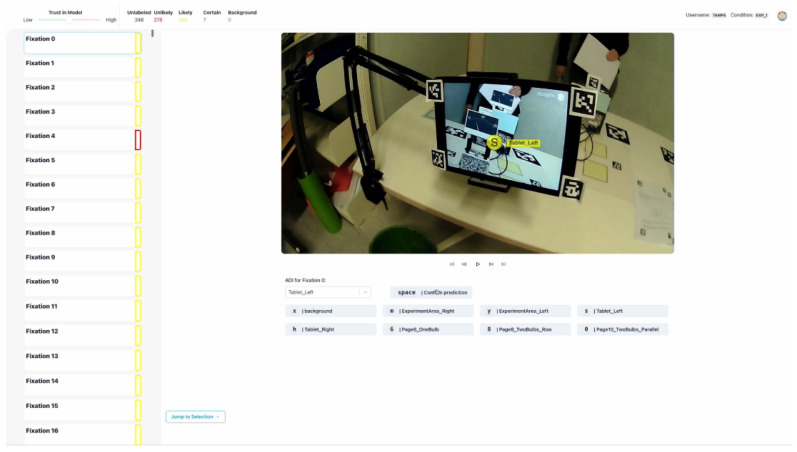
AOI suggestions.

**Figure A5:**
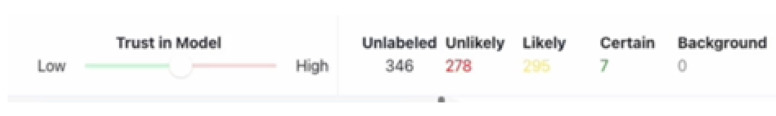
Trust-level slider.

## Data Availability

The datasets generated and/or analyzed during this study are not publicly available due to data privacy concerns related to the collection of information from children and the fact that publication was not an intended purpose. However, the data are available from the corresponding author on reasonable request.

## Abbreviations

The following abbreviations are used in this manuscript:

AOI	Area of Interest
IML	Interactive Machine Learning
AR	Augmented Reality
STEM	Science, Technology, Engineering, and Mathematics
SIFT	Scale-Invariant Feature Transform
SVC	Support Vector Classification
VIA	VGG Image Annotator
CVAT	Computer Vision Annotation Tool
FRNet	Feature Map Reconstruction Network
CNN	Convolutional Neural Network
SUS	System Usability Scale
SSI	Semi-Structured Interview
SGD	Stochastic Gradient Descent
ROC	Receiver Operating Characteristic
AUC	Area Under the Curve
FSL	Few-Shot Learning
exp_1	Experiment Phase 1
exp_2	Experiment Phase 2
E	Experiment_Area (left and right)
E_L	Experiment_Area_Left
E_R	Experiment_Area_Right
T	Tablet (left and right)
T_L	Tablet_Left
T_R	Tablet_Right
P	(Workbook) Page
P_6	Page6_OneBulb
P_8	Page8_TwoBulbs
P_10	Page10_TwoBulbs_Parallel
BG	Background

## Appendix A. Interview Guide

We conducted a semi-structured interview to understand how the IML-support version of eyeNotate was used and how that might impact the efficiency and validity of the annotation process. In the following, we outline the interview process and the instructions provided to participants. Instructions and information for participants are marked as quotes (translated into English).

### Appendix A.1. General Information

We introduced the purpose of the interview and explained the goal, i.e., to gather participants’ feedback and insights about their experience using the annotation tool:

“Thank you for participating in our study. The purpose of this interview is to gather your feedback and insights about your experience using the annotation tool. Your responses will help us understand how well the tool worked for you and will provide valuable insights that we can use to improve the tool in the future. During the interview, I will ask you a series of open-ended questions about your experience using the tool. There are no right or wrong answers, and we are interested in hearing your honest opinions and perspectives. You can take your time to think about your responses, and you are welcome to ask any questions you may have. Is there anything you would like to know before we begin the interview? ”

### Appendix A.2. Questions

We asked seven questions about features of the baseline and IML-support version of the eyeNotate annotation tool. We showed participants a screenshot or short video to better recall its functionality.

#### Appendix A.2.1. Q1—Fixation Selection

Question to assess how participants selected fixations. This can help to understand the thought processes and decision-making strategies they use.

- “In our annotation tool, there are several ways you can select fixations. One option is to use the mouse to scroll through the fixation list on the left side of the screen. Alternatively, you can use the forward and back buttons in the video player to move through the data and locate the fixation you want to annotate. Lastly, you can use the arrow key shortcuts to navigate through the fixation list.”
- “Can you describe the process you typically used to select fixations for annotations?”

Follow-up questions:

- “Can you explain why you chose to use the particular method of selection that you described?”
- “Were there any specific factors or criteria that influenced your decision to use that method?”
- “Did you encounter any challenges or difficulties when using that method of selection?”

#### Appendix A.2.2. Q2—Fixation Annotation

Question to assess how participants were using the tool to annotate the data.

“Can you describe the typical steps you took to annotate a fixation?”

Follow-up questions:

- “Can you explain why you chose to use the particular method of annotation that you described?”
- “Were there any specific factors or criteria that influenced your decision to use that method?”
- “Did you encounter any challenges or difficulties when using that method of annotation?”

#### Appendix A.2.3. Q3—Visual Annotation Feedback

Question to assess the role that visualizations played in the annotation process.

- “When you annotate a fixation using the annotation tools, you will receive visual feedback to confirm your annotation. In the video view, the fixation will be highlighted in green, and a label with your annotation will appear next to the fixation point. In the fixation list, the corresponding fixation will also be marked with a green indicator to show that it has been annotated.”
- “Can you describe how visualizations influenced your decisions about which annotations to create?”

Follow-up questions:

- “Which visualizations did you find the most helpful in making your annotation decisions? Why?”
- “Did you use any of the visualizations to check the correctness of your annotations? If so, which ones and why?”
- “Can you describe how you used the visual feedback to monitor your progress during the annotation process?”
- “Did you follow a specific strategy while annotating the fixations? If so, can you describe your strategy?”
- “Did your strategy change during the annotation process? If so, can you explain why and how it changed?”

#### Appendix A.2.4. Q4—General Experience (Baseline)

Questions were designed to assess the participants’ overall impressions of the tool and retrieve their suggestions for improvements. We asked open-ended questions to encourage the participants to reflect on their experiences and provide feedback so we could gather valuable insights into the usability and effectiveness of the tool.

- “Can you describe your overall experience with the tool?”
- “What did you like the most?”
- “What did you like the least?”
- “Did you encounter any challenges or difficulties while using the tool? If so, can you describe them and how you overcame them?”
- “Do you think the tool was easy to use and understand? Why or why not?”

#### Appendix A.2.5. Q5—AOI Suggestions

These questions were designed to gather detailed information about the participants’ use of the AOI suggestions and their perceptions of their accuracy and usefulness.

“The tool provides you with suggestions for annotation based on the confidence level of the prediction. These suggestions can be classified as having high, medium, or low confidence, and are indicated by the color of the suggestion (e.g., green for high confidence, yellow for medium confidence, red for low confidence).”

Follow-up questions:

- “Can you describe how you used the suggestions for AOIs while annotating the fixations?”
- “Now we want to know how often did you accept or reject the suggestions. Please estimate your acceptance rates on a scale of 1–10, where one is low and 10 is high.”
- “How accurate do you think the AI suggestions were? Please rate their accuracy on a scale of 1–10, where one is low and 10 is high.”
- “Did the AOI suggestions help you in the annotation process?”
- “If so, how and in what ways? If not, why do you think that was the case?”

#### Appendix A.2.6. Q6—Trust-Level Slider

These questions were designed to gather detailed information about the participants’ use of the trust-level slider and their perceptions of its usefulness and effectiveness.

“The tool includes a trust-level slider that allows you to adjust the level of support you receive from the tool’s automatic suggestions. By moving the slider to the left or right, you can increase or decrease the confidence threshold for the suggestions, respectively.”

Follow-up questions:

- “Can you describe how you used the trust-level slider?”
- “In your own words, can you explain the effects of moving the slider to the left or right?”
- “Did you consider the trust-level slider to be a useful feature? Why or why not?”
- “Are there any improvements or changes you would suggest for the trust-level slider? If so, what are they and why do you think they would be beneficial?”

#### Appendix A.2.7. Q7—General Impression (IML-Support)

These questions were designed to gather the participants’ overall impressions of the tool and its AI support and their suggestions for improvements.

- “Can you describe your overall experience with the tool?”
- “What did you like the most?”
- “What did you like the least?”
- “Did you encounter any challenges or difficulties while using the tool? If so, can you describe them and how you overcame them?”
- “Are there any features or improvements you would like to see in the tool? If so, what are they and why do you think they would be beneficial?”
